# Circulating IL-17A accumulation reflects effective target blockade with secukinumab in spondyloarthritis

**DOI:** 10.3389/fimmu.2026.1738435

**Published:** 2026-02-26

**Authors:** Julio Manuel Martínez-Moreno, Adrián Llamas-Urbano, Laura Romero-Zurita, Yas Hanaee, Alejandro Escudero-Contreras, Peter Blake, Keith Rawson, Eduardo Collantes-Estevez, Nuria Barbarroja, Clementina López-Medina, Carlos Pérez-Sánchez

**Affiliations:** 1 Maimonides Biomedical Research Institute of Cordoba (IMIBIC), Reina Sofia Hospital, University of Cordoba, Cordóba, Spain; 2 Cobiomic Bioscience SL, EBT UCO/IMIBIC, Cordóba, Spain; 3 Department of Cell Biology, Immunology and Physiology, Agrifood Campus of International Excellence, University of Córdoba, Córdoba, Spain

**Keywords:** biomarkers, IL-17A, proximity extension assay, secukinumab, spondylarthritis, therapy response

## Abstract

**Objectives:**

To investigate the dynamics of IL-17A levels in patients with spondyloarthritis (SpA) treated with secukinumab and to determine the relationship between circulating IL-17A forms and clinical response.

**Methods:**

We analyzed serum IL-17A levels in 33 samples from 11 SpA patients using the highly sensitive proximity extension assay (PEA) at baseline and after 6 and 12 months of secukinumab treatment. Clinical parameters including ASDAS and CRP were recorded. To distinguish free IL- 17A from secukinumab-bound IL-17A, we developed an innovative IgG column-based assay that separates free cytokine from antibody-conjugated fractions.

**Results:**

Serum IL-17A levels significantly increased at 6 and 12 months post-secukinumab treatment. This increase correlated negatively with ASDAS and CRP, indicating better clinical response. Analysis showed that the rise was mainly due to the secukinumab-bound IL-17A fraction, while free IL-17A levels remained stable.

**Conclusion:**

Our study reveals that the elevated IL-17A levels detected after secukinumab treatment primarily represent the antibody-conjugated form rather than free cytokine. This conjugated fraction is associated with improved clinical outcomes, suggesting it could serve as a biomarker for therapeutic efficacy. The novel IgG column-based assay provides a valuable tool for differentiating cytokine forms in patients undergoing monoclonal antibody therapies, with potential applications beyond SpA. These findings advance understanding of IL-17A dynamics during treatment and open new avenues for personalized monitoring and management in autoimmune diseases.

## Highlights

- IL-17A levels increase after secukinumab treatment and correlate with improved clinical response in SpA patients.
- Elevated IL-17A levels after treatment predominantly represent antibody-bound cytokine rather than free IL-17A, thereby reflecting effective target blockade.
- Novel IgG-based assay enables differentiation of free and bound cytokines, aiding personalized therapy monitoring.

## Introduction

IL-17A is a pivotal pro-inflammatory cytokine involved in the immunopathogenesis of spondyloarthritis (SpA), playing a critical role in driving inflammation and tissue damage. Due to its central involvement in disease mechanisms, IL-17A has become a major therapeutic target in SpA and related inflammatory conditions (1, 2). Secukinumab, a fully human monoclonal IgG1 antibody that binds IL-17A with high affinity and specificity, effectively neutralizes its activity and has demonstrated substantial efficacy in reducing disease activity and improving clinical outcomes in patients with SpA (3). Despite its clinical success, the biological effects of secukinumab on circulating IL-17A levels remain incompletely understood.

Interestingly, several studies have paradoxically reported increased circulating IL-17A levels following secukinumab treatment primarily in patients with psoriasis (4–7). This phenomenon has raised important questions regarding the nature of the detected cytokine—whether it represents biologically active free IL-17A or IL-17A bound to the therapeutic antibody, which might affect its bioavailability and function. To date, these studies have not examined the relationship between elevated IL-17A levels and clinical response, nor have they experimentally distinguished between free and antibody-bound cytokine forms.

In our study, we addressed these gaps by developing a novel assay that isolates total IgG from patient serum to specifically measure IL-17A bound to secukinumab, enabling differentiation from free IL-17A. Using this approach, we observed that the increase in IL-17A following treatment primarily reflects the antibody-conjugated fraction rather than the free form. Moreover, we demonstrated that this increase in total IL-17A correlates with improved clinical outcomes, providing new insights into cytokine dynamics during IL-17A blockade. These findings have important implications for the development of biomarkers to monitor therapeutic efficacy and optimize personalized treatment strategies in SpA.

## Methods

We analyzed IL-17A levels in 33 serum samples from patients with spondyloarthritis (SpA) using the Olink Target 48 Proximity Extension Assay (PEA), a highly sensitive technique uniquely suited for detecting low concentrations of inflammatory mediators (Cobiomic Bioscience SL, Cordoba, Spain). This sensitivity is achieved through dual antibody recognition linked to oligonucleotides that hybridize and are quantified by qPCR (8). IL-17A measurements were performed with a commercially validated antibody pair supplied by Olink.

The cohort included 11 SpA patients sampled prior to secukinumab treatment and again at 6- and 12-months following therapy initiation. All patients provided informed consent, and the study was approved by the Ethics Committee of Córdoba Reina Sofía Hospital. The research adhered to the principles outlined in the Declaration of Helsinki.

**Figure 1:**
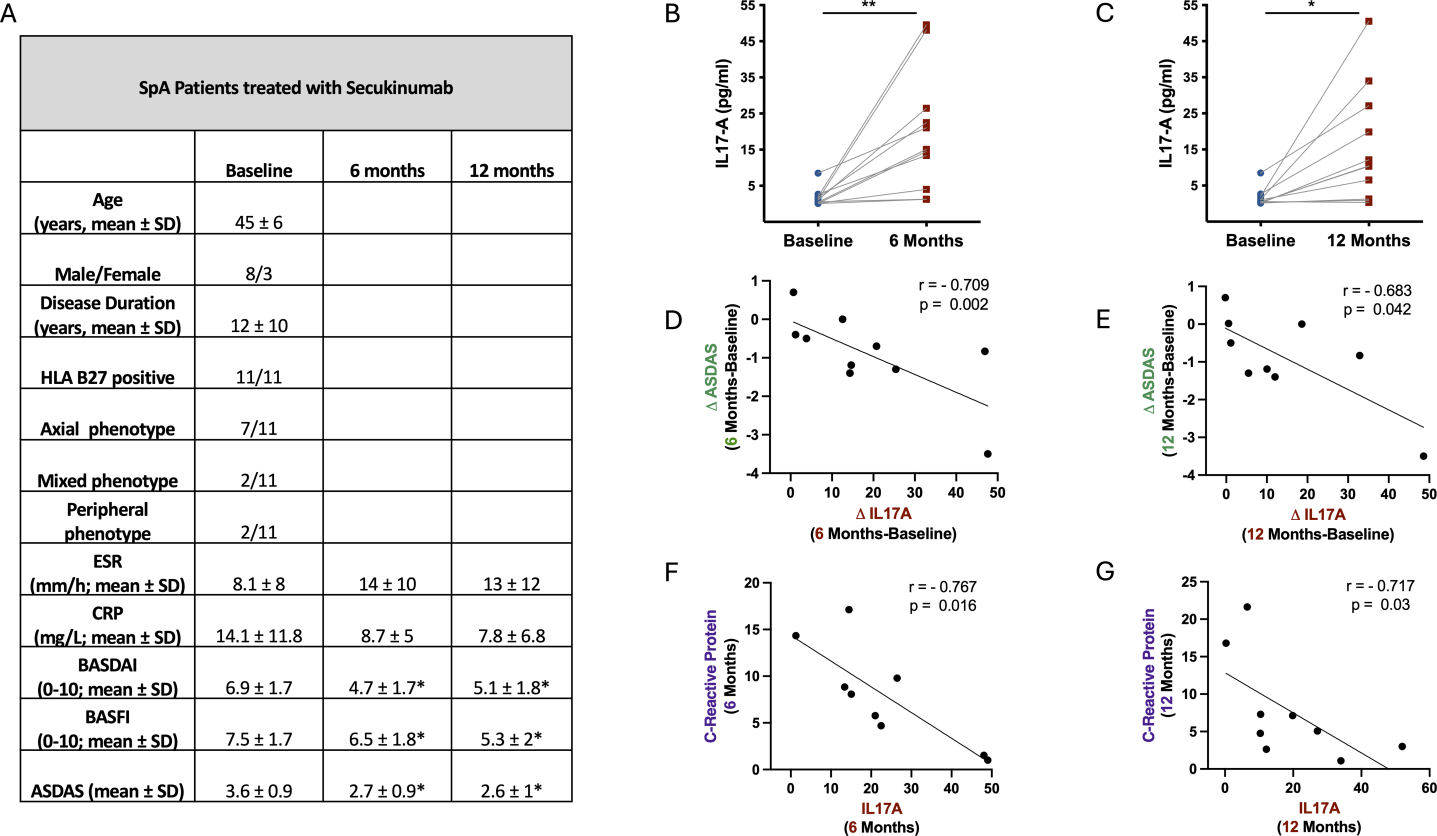
Serum IL-17A levels and their association with clinical outcomes during secukinumab treatment in SpA patients. **(A)** Clinical and demographic characteristics of the study cohort. **(B, C)** Total IL-17A levels in serum at baseline, 6 months, and 12 months after initiation of secukinumab, measured by proximity extension assay (PEA). **(D, E)** Negative correlation between changes in IL-17A levels and ASDAS scores at 6 and 12 months, respectively. **(F, G)** Negative correlation between IL-17A levels and CRP levels at 6 and 12 months, respectively. * p-value < 0.05 vs baseline. SpA, Spondyloarthritis; ESR, Erythrocyte Sedimentation Rate; CRP, C-Reactive Protein; BASDAI, Bath Ankylosing Spondylitis Disease Activity Index; BASFI, Bath Ankylosing Spondylitis Functional Index; ASDAS, Ankylosing Spondylitis Disease Activity Score; SD, standard deviation.

All patients underwent comprehensive clinical characterization, including demographics (age, sex, disease duration), Axial Spondyloarthritis Disease Activity Score (ASDAS), c-reactive protein (CRP) levels, and HLA-B27 status. Detailed clinical characteristics are provided in **Figure 1A**. Changes in IL-17A levels were correlated with changes in ASDAS and CRP after treatment.

**Figure 2:**
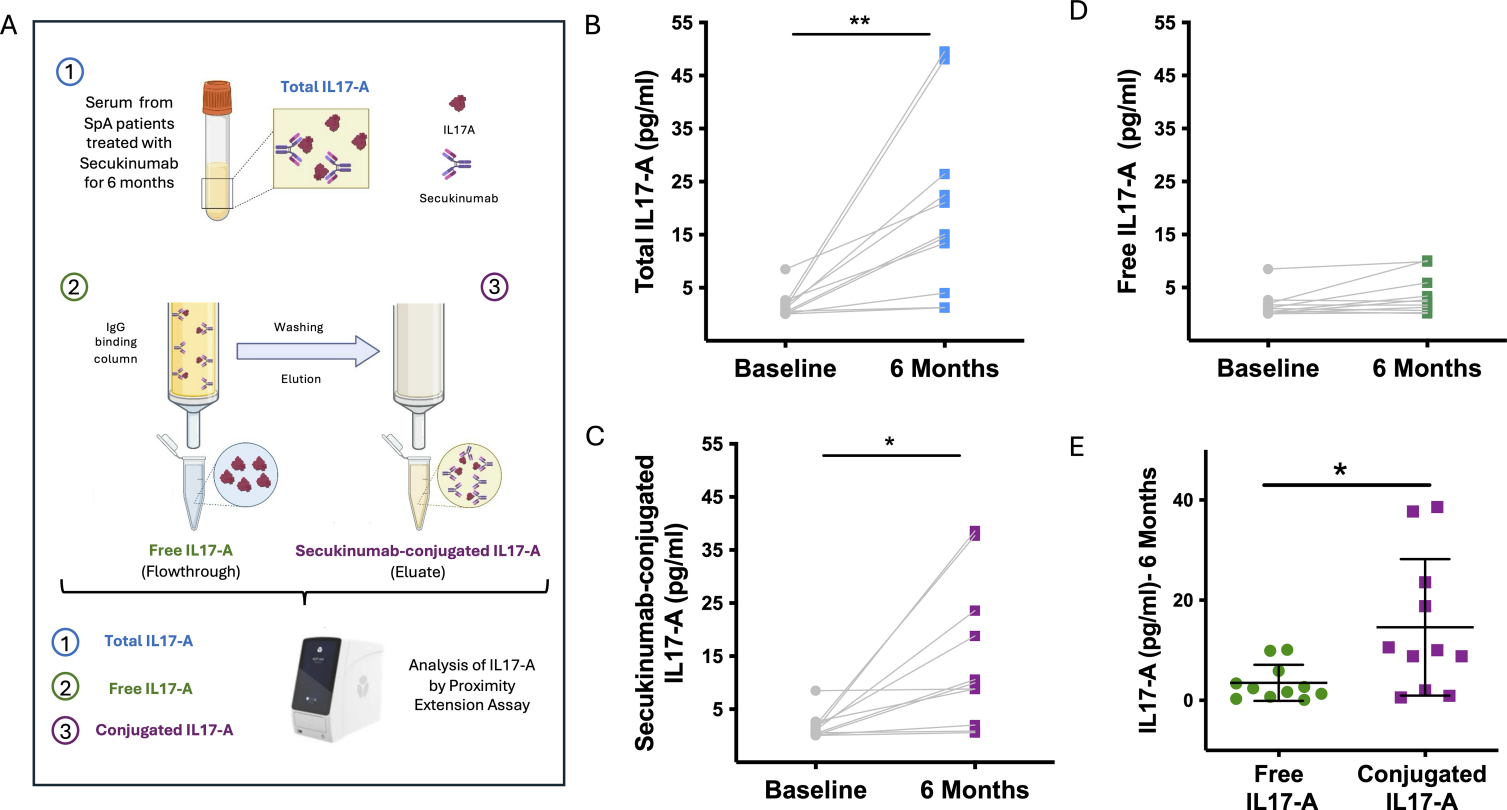
Differentiation of free and secukinumab-bound IL-17A fractions in serum of SpA patients. **(A)** Schematic workflow of the IgG column–based assay used to separate total, free, and antibody-conjugated IL-17A fractions. Serum was loaded onto IgG-binding columns; free IL-17A was collected in the flow-through, while secukinumab and secukinumab–IL-17A complexes were retained and subsequently eluted. **(B)** Total serum IL-17A levels at baseline and after 6 months of secukinumab treatment. **(C)** Secukinumab-bound IL-17A fraction significantly increased at 6 months. **(D)** Free IL-17A levels remained unchanged during treatment. **(E)** Comparison of free versus antibody-bound IL-17A at 6 months, showing higher levels of the conjugated fraction.

To distinguish between free IL-17A and IL-17A bound to secukinumab, we developed a novel assay using IgG-binding NAb™ Spin columns (0.2 mL) (ThermoFisher, USA). These columns exploit the affinity of bacterial protein G for the Fc region of IgG, thereby retaining all antibodies present in the sample. Briefly, 125 µL of serum from patients treated with secukinumab for 6 months was loaded onto the columns and incubated at room temperature with end-over-end mixing for 10 minutes. The columns captured total IgG, including both free secukinumab and secukinumab–IL-17A complexes, while the flow-through—containing only free, unbound IL-17A—was collected for analysis. After three washes with 400 µL of Binding Buffer, bound IgG and immune complexes were eluted with 400 µL of Elution Buffer into vials preloaded with 40 µL of Neutralization Buffer. IL-17A measurement in this eluate reflected secukinumab-bound IL-17A. Thus, quantification of IL-17A across the original serum, flow- through, and eluate fractions corresponded to total IL-17A, free IL-17A, and secukinumab- bound IL-17A, respectively (**Figure 2A**). The IgG depletion step was validated by independent quantification of secukinumab (EAD-Secu; RayBiotech), which was retained in the eluate and absent from the flow-through used for free IL-17A measurement.

## Results

The total levels of IL-17A in serum of SpA patients were significantly increased at both 6 and 12 months following secukinumab treatment (**Figures 1B, C**). Adjustment for age, sex, and phenotype did not significantly affect the observed changes in IL-17A. To explore the relationship between this increase and clinical response, we correlated changes in ASDAS scores with changes in IL-17A levels at both time points. We found a significant negative correlation, indicating that patients with greater increases in serum IL-17A experienced larger reductions in ASDAS scores (**Figures 1D, E**). Additionally, IL-17A levels negatively correlated with CRP levels at 6- and 12-months post-treatment (**Figures 1F, G**). These findings suggest that patients exhibiting the highest elevations in IL-17A after treatment had the lowest CRP levels, reinforcing the association between increased IL-17A detected by PEA and improved clinical outcomes.

To determine whether the observed increase in IL-17A levels was due to free or antibody- conjugated forms, we analyzed serum samples collected after 6 months of treatment. We compared total IL-17A levels with the free and secukinumab-conjugated IL-17A fractions, separated using an IgG column isolation method (**Figure 2A**). Our results showed that the significant increase in total IL-17A after 6 months (**Figure 2B**) was driven by a marked elevation in the secukinumab–IL-17A conjugated fraction (**Figure 2C**), while levels of free IL-17A remained unchanged before and after therapy (**Figure 2D**). Moreover, the amount of conjugated IL-17A after treatment was significantly higher than the free IL-17A levels post- secukinumab (**Figure 2E**).

## Discussion

Previous studies have predominantly reported paradoxical increases in circulating IL-17A levels following secukinumab treatment in patients with psoriasis (4–6). However, none of these investigations have established a direct correlation between elevated IL-17A and clinical response, nor have they elucidated whether the detected IL-17A represents free cytokine or an antibody-bound fraction. This gap in knowledge has limited our understanding of the biomarker’s clinical relevance and the mechanistic insights into cytokine dynamics during monoclonal antibody therapy.

To our knowledge, this study is the first to demonstrate a significant elevation of IL-17A levels in patients with spondyloarthritis following treatment with secukinumab. Our findings address that critical gap by demonstrating that the increased IL-17A observed after secukinumab treatment predominantly corresponds to IL-17A conjugated with the therapeutic antibody rather than free cytokine. This conjugation likely leads to prolonged circulation time and reduced clearance, explaining the elevated detection levels, although compensatory cytokine production and altered clearance cannot be entirely excluded. Importantly, we provide the first evidence linking this increase in antibody-bound IL-17A with improved clinical outcomes, suggesting that the presence of conjugated IL-17A may serve as a proxy for effective cytokine blockade. A key factor enabling these insights is the use of the Olink proximity extension assay (PEA), whose exceptional sensitivity and specificity allow for precise quantification of low-abundance inflammatory mediators (9). Notably, a previous study utilizing the same PEA technology also reported an elevation of IL-17A levels following secukinumab treatment in patients with psoriasis, supporting the robustness of this approach (10). Traditional immunoassays such as ELISA or Luminex may fail to discriminate between free and antibody-bound cytokine or lack the sensitivity to detect subtle but clinically relevant changes (11, 12). The ability to measure these distinct IL-17A fractions not only enhances our understanding of drug-target interactions but also opens avenues for developing predictive biomarkers of therapeutic response.

Moreover, our development of a novel IgG column-based assay to separate free and antibody- conjugated cytokines represents a significant technological advance with broad implications. This methodology can be adapted for studies involving other monoclonal antibodies, particularly IgG isotypes, across a wide range of diseases. By enabling differentiation between free and bound cytokines, this approach facilitates more nuanced pharmacodynamic assessments, potentially improving patient stratification and personalized treatment strategies.

From a clinical perspective, monitoring conjugated IL-17A levels may serve as an innovative biomarker to identify patients who truly benefit from secukinumab therapy. Patients exhibiting higher levels of IL-17A bound to the antibody are likely those in whom the cytokine is effectively neutralized, correlating with better disease control. This could guide treatment decisions, optimize therapeutic regimens, and avoid unnecessary exposure to ineffective therapies.

While the sample size is relatively small, the consistency of the results across analyses supports the robustness of the findings. In conclusion, our study helps to clarify the biological interpretation of increased circulating IL-17A levels following secukinumab treatment, supporting their relevance as a readout of target engagement rather than direct cytokine activity. In addition, we present a sensitive technological approach and conceptual framework that may be useful for biomarker development in immunomodulatory therapies. Future studies in larger cohorts and across different monoclonal antibody treatments will be required to further define the clinical utility and broader applicability of this strategy in precision medicine.

## Data Availability

The raw data supporting the conclusions of this article will be made available by the authors, without undue reservation.
